# Generation of dendritic cell-based vaccine using high hydrostatic pressure for non-small cell lung cancer immunotherapy

**DOI:** 10.1371/journal.pone.0171539

**Published:** 2017-02-10

**Authors:** Nada Hradilova, Lenka Sadilkova, Ondrej Palata, Dagmar Mysikova, Hana Mrazkova, Robert Lischke, Radek Spisek, Irena Adkins

**Affiliations:** 1 SOTIO, Prague, Czech Republic; 2 Department of Immunology, 2nd Faculty of Medicine, Charles University and University Hospital Motol, Prague, Czech Republic; 3 Thoracic and Lung Transplantation Division, 3rd Department of Surgery, 1st Faculty of Medicine, Charles University and University Hospital Motol, Prague, Czech Republic; Mie University Graduate School of Medicine, JAPAN

## Abstract

High hydrostatic pressure (HHP) induces immunogenic death of tumor cells which confer protective anti-tumor immunity *in vivo*. Moreover, DC pulsed with HHP-treated tumor cells induced therapeutic effect in mouse cancer model. In this study, we tested the immunogenicity, stability and T cell stimulatory activity of human monocyte-derived dendritic cell (DC)-based HHP lung cancer vaccine generated in GMP compliant serum free medium using HHP 250 MPa. DC pulsed with HHP-killed lung cancer cells and poly(I:C) enhanced DC maturation, chemotactic migration and production of pro-inflammatory cytokines after 24h. Moreover, DC-based HHP lung cancer vaccine showed functional plasticity after transfer into serum-containing media and stimulation with LPS or CD40L after additional 24h. LPS and CD40L stimulation further differentially enhanced the expression of costimulatory molecules and production of IL-12p70. DC-based HHP lung cancer vaccine decreased the number of CD4^+^CD25^+^Foxp3^+^ T regulatory cells and stimulated IFN-γ-producing tumor antigen-specific CD4^+^ and CD8^+^ T cells from non-small cell lung cancer (NSCLC) patients. Tumor antigen specific CD8^+^ and CD4^+^ T cell responses were detected in NSCLC patient’s against a selected tumor antigens expressed by lung cancer cell lines used for the vaccine generation. We also showed for the first time that protein antigen from HHP-killed lung cancer cells is processed and presented by DC to CD8^+^ T cells. Our results represent important preclinical data for ongoing NSCLC Phase I/II clinical trial using DC-based active cellular immunotherapy (DCVAC/LuCa) in combination with chemotherapy and immune enhancers.

## Introduction

Lung carcinoma represents the leading cause of cancer mortality worldwide with dismal prognosis as the majority of patients are diagnosed in an advanced stage [1]. This disease can be divided into non-small cell lung carcinoma (NSCLC) (80–85%) and small cell carcinoma (15–20%). NSCLC is represented by three major histological subtypes such as adenocarcinoma (50%), squamous cell carcinoma (30%) and large cell carcinoma (5%). Despite advances in surgery, chemotherapy and irradiation the 5-year survival rate has only increased from 5% to 15% over the last 25 years [2]. Therefore the development of new therapeutic strategies in treatment of lung carcinoma is essential. DCs play a key role in inducing and shaping of immune responses and have proven to be crucial in inducing anti-tumor immunity [3, 4]. Since the first DC-based clinical trial in 1995 [5], the technique of *ex vivo* DC manufacturing have developed leading to a large-scale production which conforms to strict regulatory agencies and Good Manufacturing Practices (GMP) requirements. The success of DC-based cancer immunotherapy was documented by FDA approval of Sipuleucel-T (Provenge) for treatment of patients with asymptomatic or minimally symptomatic metastatic castration-resistant prostate cancer in 2010 [6]. Contrary to other malignancies, there are little data on DC-based immunotherapy of lung carcinoma in patients [3]. Several phase I studies for NSCLC treatment were conducted over the past 10 years using DC generated according to various protocols and loaded with TAA-derived peptides [7–10], proteins [11] or tumor cell lysate [12–14, 2]. Surprisingly, only one research group used irradiated and UVB-treated allogeneic whole tumor cells to generate DC-based lung cancer vaccine [15–18]. These studies proved that DC-based lung cancer immunotherapy is safe and well tolerated and in some patients clinical benefit was observed [12, 13, 8, 7, 19]. There are only two studies published so far that have documented prolonged overall survival of NSCLC patients [14, 10].

The success of DC-based cancer immunotherapy depends on the range of TAA presented by DC and the capacity of DC to produce cytokines such as IL-12p70 and provide costimulation to T cells [3]. Several cancer chemotherapeutics and cell death-inducing physical modalities have been described to induce immunogenic cell death (ICD) of tumor cells [20, 21]. Tumor cell ICD is characterized by induction of endoplasmic reticulum stress response, production of reactive oxygen species and surface exposure/emission of danger-associated molecules such as calreticulin, heat shock proteins, HMGB1 or ATP [22, 20, 23]. Tumor cells undergoing ICD activate various immune cells including DC to stimulate anti-tumor immune responses [20, 23]. We have previously shown that the application of high hydrostatic pressure (HHP) on human cancer cell lines and primary tumor cells induces ICD [24]. Human monocyte-derived DC pulsed with HHP-killed tumor cells displayed increased expression of maturation-associated molecules and pro-inflammatory cytokine production which resulted in stimulation of IFN-γ-producing CD8^+^ and CD4^+^ T cells *in vitro* [24]. Moreover, DC loaded with HHP-treated tumor TC-1 or prostate tumor cells TRAMP-C2 combined with docetaxel chemotherapy significantly inhibited growth of tumors in mouse models [25].

In this study, we describe the generation of human DC-based lung cancer vaccine in serum free GMP-compliant media X-VIVO 15 using HHP-killed lung cancer cell lines H520 and H522 as source of TAA and poly(I:C) as a DC maturation signal. DC-based HHP lung cancer vaccine exhibited functional plasticity after additional stimulation in serum containing medium with LPS or CD40L and was fully competent to stimulate CD8^+^ and CD4^+^ T cells. Moreover, DC-based HHP vaccine generated from NSCLC patients induced tumor antigen-specific CD4^+^ and CD8^+^ T cell responses *in vitro*. CD8^+^ and CD4^+^ T cells specific to selected tumor antigens expressed in H520 and H522 were detected in NSCLC cancer patients. Importantly, we showed here for the first time that DC process and present protein antigen from HHP-killed tumor cells to induce antigen-specific CD8^+^ T cells. These data represent preclinical data for ongoing phase I/II NSCLC clinical trial combining DC-based active cellular immunotherapy with chemotherapy and immune enhancers (NCT02470468).

## Methods

### Non-small cancer cell lines

The non-small cell lung cancer cell lines H520 (squamous cell carcinoma), H522 and A549 (adenocarcinoma) were obtained from the American Type Culture Collection (Manassas, USA). H520 and H522 were cultivated at 37°C in a humidified atmosphere containing 5% CO_2_ in RPMI-1640 complete medium (Gibco) supplemented with 10% heat-inactivated fetal bovine serum (PAA), 2 mM GlutaMAX I CTS (Gibco) and 100 U/ml penicillin + 100 mg/ml streptomycin (Gibco). A549 cells were grown in F12 medium (Gibco) supplemented as described above.

### Preparation of HHP-killed cancer cells

2 × 10^6^ lung cancer cells were killed with high hydrostatic pressure (HHP) of 250 MPa for 10 minutes using a custom-made device (Resato International BV, Netherlands) located in the GMP manufacturing facility of Sotio, Prague, Czech Republic. After the HHP treatment cells were further incubated at 37°C for 2 h. Cells were collected by centrifugation (1500 rpm, 5 min), resuspended in 1 ml of CryoStor™ CS-10 (BioLife Solution) and stored at -80°C for 24h. HHP-killed cells were thawed and washed twice with serum free media X-VIVO 15 (supplemented with recombinant transferrin, Lonza) before addition to DC. The induction of cell death and the exposure of immunogenic molecules (HSP70, HSP90 and calreticulin) by HHP were determined by flow cytometry (S1 Fig).

### Generation of DC-based HHP lung cancer vaccine

Peripheral blood mononuclear cells (PBMC) were obtained from buffy coats of healthy donors by Ficoll-Paque gradient centrifugation (GE Healthcare). 75 × 10^6^ PBMCs/flask in 10 ml of serum free GMP-compliant media X-VIVO 15 (supplemented with transferrin, Lonza) were plated in 75 cm^2^ culture flasks (Nunc) at 37°C and monocytes were allowed to adhere to the flask bottom for 2 h. The non-adherent fraction containing lymphocytes was collected and stored short-term at -80°C. Monocyte-derived DC were generated in X-VIVO 15 for 4 days [26] in the presence of 500 IU/ml of GM-CSF and 20 ng/ml of IL-4 (both from Gentaur). To generate DC-based HHP lung cancer vaccine, 2 × 10^5^ immature DC/well seeded in 96-well plate (Nunc) were incubated with an equal mixture (1 H520:1 H522) of thawed HHP-killed lung cancer cell lines at a DC/tumor cells ratio of 5:1 for 4 h. After that poly(I:C) (25 μg/ml, VacciGrade^TM^ InvivoGen) was added for additional 20 h.

### Phenotype and cytokine production of DC-based HHP lung cancer vaccine

DC maturation after pulsation with HHP-killed cancer cells alone or after pulsation with HHP-killed cancer cells and poly(I:C) (DC-based HHP lung cancer vaccine) was evaluated by flow cytometry. DC phenotype was determined by staining with CD80-FITC, CD86-PE, CD83-PE-Cy5.5 (Beckman Coulter), CCR7 (CD197)-APC-eFluor780 (eBioscience), CD11c-APC (Exbio) and HLA-DR-PE-Cy7 (BD Biosciences) for 20 min. Cells viability was detected with DAPI staining. CD11c^+^DAPI negative DC were analyzed for mean fluorescence intensity (MFI) of the particular maturation marker. To determine cytokine production, cell supernatants were harvested 24 h after DC’s stimulation and stored at -80°C. IL-12p70, IL-10, IFN-α and TNF-α were determined using Luminex assay (MILLIPLEX™ Human Cytokine/Chemokine Kit, Merck Millipore) by Luminex 2000 (Luminex).

### Migration and phagocytic capacity of DC-based HHP lung cancer vaccine

Migratory capacity of DC loaded with HHP-killed cancer cells alone or loaded with HHP-killed cancer cells and stimulated with poly(I:C) (DC-based HHP lung cancer vaccine) toward chemokines CCL19 and CCL21 was assessed 24 h after maturation using transwell assays (5.0 μm pore size; Costar, Corning). Lower chambers of the transwell plate were filled with 200 μl of RPMI 1640 containing 10% AB human serum with or without chemokines CCL19 (50 ng/ml) and CCL21 (50 ng/ml). 3 × 10^5^ DC in 70 μl of RPMI 1640 containing 10% AB human serum was seeded in the upper chamber of the transwell plate in duplicates. Cells were incubated at 37°C for 5 h. After that transmigrated DC in the lower chambers were harvested, stained with CD11c-APC antibody (Exbio) and counted using flow cytometry. For phagocytic assays, 1 × 10^6^ DC were stained with 2.5 μl/ml VybrantVR DiO cell labeling solution (Invitrogen) for 20 min at 37°C before pulsation with HHP-killed cancer cells. Similarly, 1 × 10^6^ thawed HHP-killed lung cancer cells were stained with 2.5 μl/ml VybrantVR DiD cell labeling solution (Invitrogen) in serum free media at 37°C for 20 min. Cells were washed twice, mixed at DC/tumor cell ratio of 1:5 and incubated for 4 h at 37°C before poly(I:C) was added to some samples for additional 20 h. Cells incubated at 4°C served as a negative control for phagocytosis. The phagocytic ability of DC was evaluated by flow cytometry as a percentage of double positive (DiO^+^DiD^+^) cells.

### Stimulation of DC-based HHP lung cancer vaccine in human serum containing media

2 × 10^5^/well of immature DC or DC pulsed with HHP-killed lung cancer cells and stimulated with poly(I:C) were left in serum-free medium X-VIVO 15, or placed into RPMI 1640 medium containing 10% human AB serum (Invitrogen), or stimulated with 1 μg/ml of LPS (*E*.*coli*, Sigma Aldrich) or placed onto a layer of CD40L-expressing A549 cells for additional 24 h. Stable CD40L-transfected A549 cells were generated by a single cell clone dilution from hygromycin resistant cells transfected with pCMV/hygro-CD40L vector (Sino Biological Inc.). Non-transfected A549 cells were used as a negative control of stimulation. DC maturation was assessed as described above by flow cytometry. IL-10 and IL-12p70 production was determined by ELISA (R&D System) according to manufacturer’s instructions.

### Generation of DC-based HHP lung cancer vaccine from PBMC of NSCLC patients

Two tubes of peripheral blood (VACUETTE® 9 ml K3 EDTA) were obtained from 6 of NSCLC patients undergoing neoadjuvant surgery in University Hospital Motol, Prague after signing an informed consent. Informed consent was obtained after the nature and possible consequences of the studies had been fully explained. The experiments were performed with an agreement of local ethical committee (University Hospital Motol, Prague, Czech Republic). PBMC were isolated by Ficoll-Pague gradient centrifugation. One half of isolated PBMC was stored at -80°C for T cell restimulation. Monocytes from the remaining PBMC were separated using CD14^+^ isolation kit (EasySep™, Human CD14 Positive Selection Kit) according to manufacturer’s instructions. 2.5 × 10^6^/well CD14^+^ cells were seeded into the 6-well plate (Nunclon) in 4 ml of X-VIVO 15 medium supplemented with GM-CSF and IL-4 as described above. On day 4, DC were pulsed with an equal mixture (1:1) of thawed HHP-killed H520 and H522 cell lines for 4 h before poly(I:C) was added for additional 20 h.

### Induction of tumor-antigen specific T cells and CD4^+^CD25^+^Foxp3^+^ T regulatory cells

2 × 10^5^/well of immature DC, poly(I:C)-treated DC, DC pulsed with HHP-killed cancer cells alone or DC pulsed with HHP-killed cancer cells and stimulated with poly(I:C) were mixed with thawed autologous lymphocytes obtained from PBMC of healthy donors and NSCLC patients at a ratio of 1 DC: 5 T cells in 96-round well plate. Cells were cultivated in RPMI-1640 medium containing 10% AB human serum (Invitrogen), 2 mM GlutaMAX I CTS (Gibco), 100 U/ml penicillin, 100 mg/ml streptomycin (Gibco), 1% non-essential amino acids (Gibco), 1 mM natrium pyruvate (Gibco) and 50 μM β-mercaptoethanol (Gibco) for 7 days. A 20 U/ml of IL-2 (PeproTech) was added on days 3 and 5 of cultivation. Poly(I:C) stimulated DC loaded with a mixture of MHC class I-restricted overlapping antigenic peptides to CMV, EBV and influenza (CEF; 2 μg/ml JPT Peptide Technologies) was used as a positive control of CD8^+^ T cell stimulation. On day 7, T lymphocytes were restimulated with freshly prepared sample-corresponding DC at the same ratio for 1 h before addition of Brefeldin A (BioLegend, 1000x). After 3 h the frequency of IFN-γ-producing and proliferating (Ki-67^+^) CD8^+^ and CD4^+^ T cells was determined using flow cytometry. Briefly, T cell were stained with CD3-Alexa 700 (Exbio), CD4-PE-Cy7 (eBioscience) and CD8-PE-Dy590 antibodies (Exbio) for 30 min. Cells were then fixed using Fixation Buffer (eBioscience), permeabilized with Permeabilization Buffer (eBioscience) and stained intracellularly with IFN-γ-FITC (BD Biosciences) and Ki67-PE (Biolegend) antibodies for 30 min. In experiments analyzing NSCLC patient’s T cell responses cells were stained extracellularly with CD3-PerCP-Cy5.5 (eBioscience), CD4-PE-Cy7 (eBioscience) and CD8-PE-Dy590 antibodies (Exbio), and intracellularly with IFN-γ-FITC (BD Biosciences) and Ki-67-Alexa 700 (BD Biosciences) antibodies. The percentage of CD4^+^CD25^+^FoxP3^+^ T regulatory cells was determined without restimulation with fresh DC by flow cytometry at day 7. T cells were stained with CD4-PE-Cy7 (eBioscience), CD8-PE-Dy590 antibodies (Exbio) and CD25-PerCP-Cy5.5 (BioLegend) for 30 min. Cells were then fixed and permeabilized as described above and stained intracellularly with Foxp3-Alexa Fluor 488 (eBiosciences) for 30 min.

### Generation of matrix protein 1-expressing A549 cells and detection of MP1_58-66_–specific CD8^+^ T cells

Shorter version of M1 gene (M1v1–699 bp) was obtained by PCR from a plasmid pCMV-H7N9-AH-13-M1 (Sino Biologicals Inc.) bearing a coding sequence (756 bp) for full-length matrix protein 1 (MP1) from H7N9 virus. M1v1 sequence was inserted into pcDNA-YFP plasmid (Invitrogen) to obtain pcDNA3-M1v1-YFP. A549 cells grown to 70% confluence in 24-well plate were transfected with 1 μg pcDNA3-M1v1-YFP (linearized by FspI restriction endonuclease) using Lipofectamin RNAiMAX (Thermo Fisher scientific) in 0.5 ml serum free F12 medium and incubated overnight at 37°C. Then the cell medium was replaced by serum containing F12 medium supplemented with 300 μg/ml of hygromycin B (Sigma-Aldrich). After 7 days the YFP fluorescence of transfected cells was detected by flow cytometry. Positive cells were diluted 1–3 cells/well into 96-well plate and cultivated under hygromycin B selection for 3 weeks. Autologous T cells were stimulated by HLA-A*201^+^ immature DC, poly(I:C)-treated DC or DC-based HHP lung cancer vaccine generated with thawed HHP-killed MP1-expressing A549 and poly(I:C) as described above. DC incubated with poly(I:C) and MP1_58-66_ (GILGFVFTL) (10 μg/ml; MBL International) were used as a positive control for CD8^+^ T cell stimulation. After 8 days, MP1_58-66_ specific CD8^+^ T cells were stained with MP1_58-66_-HLA-A*201 Tetramer-PE (2 μl/sample; MBL International) for 30 min together with CD3-PerCP-Cy5.5 (eBioscience), CD4-PE-Cy7 (eBioscience) and CD8-Alexa Fluor 700 (Exbio) antibodies and analyzed by flow cytometry.

### Detection of tumor antigen-specific T cells in NSCLC patients

PBMC were isolated and cryopreserved in liquid nitrogen from 36 NSCLC patients undergoing neoadjuvant surgery in University Hospital Motol, Prague in 2014–2016 after signing an informed consent. The experiments were performed with an agreement of local ethical committee (University Hospital Motol, Prague, Czech Republic). PBMC were thawed and seeded at 2 x 10^5^/well in 100 ul medium containing AB serum as described above and incubated overnight at 37°C. Then tumor antigen specific pepmixes containing 33–128 peptides from MAGE-A3, MAGE-A4, hTERT, Survivin and Mucin-1 proteins (JPT Peptide Technologies) were added according to manufacturer’s recommendation at concentration of 10 ug/ml and incubated for 9 days. A mixture of mainly MHC class I-restricted overlapping antigenic peptides to CMV, EBV and influenza [CEF] (PM-CEF-E; 2 μg/ml JPT Peptide Technologies) and mainly MHC class II-restricted peptides to influenza virus Hemagglutinin [HA] (PM-INFA-HA/Cal; 10 μg/ml JPT Peptide Technologies) were used as positive controls of CD8^+^ T cell and CD4^+^ T cell stimulation, respectively. 20 U/ml of IL-2 (PeproTech) was added on days 4 and 7 of cultivation. Cells were restimulated with the peptides on day 9 for 2h. Then Brefeldin A (BioLegend, 1000x) was added and cells were incubated overnight. The production of IFN-γ from CD8^+^ and CD4^+^ T cells was detected by flow cytometry as described above using CD3-AlexaFluor700, IFN-γ-PE-Cy7, CD4-PerCP-Cy5.5, CD8a-eFluor450 (BioLegend) antibodies. The viability of IFN-γ-producing T cells was stained with LIVE/DEAD Fixable Aqua Dead Stain kit (405 nm excitation) (Invitrogen). The percentage of negative control (no antigen added to PBMCs) was deducted from the samples stimulated with an antigenic peptide mixture.

### qPCR analysis of antigen expression

Total RNA was isolated from cell lysates using RNeasy mini kit (Qiagene) in accordance with the manufacturer’s protocol including DNA digestion step. The RNA purity and concentration was determined spectrophotometrically using a Nanodrop 2000c (Fisher Thermo scientific). Reverse transcription was performed from 1 μg of total RNA using an iScript cDNA synthesis kit (BioRad) using probes specific to MAGE-A3, MAGE-A4, hTERT, Survivin and Mucin-1 designed, synthetized and approved by TIB MOLBIOL Syntheselabor GmBh, Germany. Expression of genes was determined by qPCR on CFX96 Touch™ Real-Time PCR Detection System (BioRad) in duplicate. The each 10 μl reaction contained 5 μl of KAPA PROBE FAST qPCR Master Mix (Kapa Biosystems), 0.5 μM of each forward and reverse primers (TIB Molbiol), 0.2 μM of TaqMan probe (TIB Molbiol), 1.5 μl of DNase-free water and 2 μl of 10x diluted cDNA. The reaction thermal protocol was following: 3 minutes at 95°C followed by 45 cycles of amplification (95°C for 15 s, 60°C for 60 s). The formation of PCR products of the expected lengths was confirmed by agarose gel electrophoresis. The Cq values were determined using CFX Manager software (BioRad) and the relative expressions of the studied genes were calculated with GenEx software (MultiD Analyses) with cut off at 36 cycle. The cut off for positive expression was set to relative expression value greater than the mean plus 2 SDs of the control non-tumoral tissue.

### Statistical analysis

Two-tailed paired t-test or unpaired, nonparametric Mann-Whitney test were applied for data analysis using GraphPad PRISM 6 (San Diego, California, USA). The results were considered statistically significant if * p < 0.05, ** p < 0.01 or *** p < 0.001.

## Results

### DC-based HHP lung cancer vaccine displays mature phenotype, produces pro-inflammatory cytokines and increases chemotactic migration

**Fig 1:**
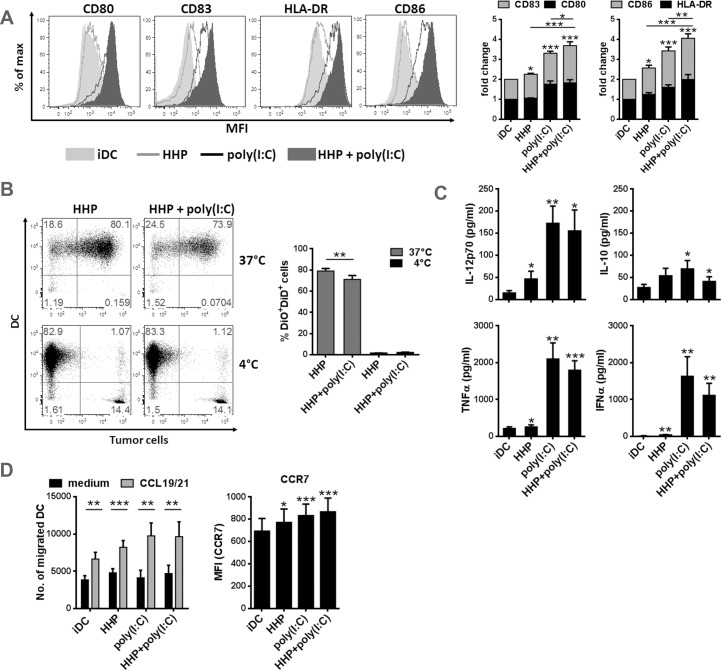
DC-based HHP lung cancer vaccine displays mature phenotype, produces pro-inflammatory cytokines and increases chemotactic migration. 2 × 10^5^ monocyte-derived DC generated in serum-free media X-VIVO 15 were left untreated [iDC] pulsed with thawed 4 × 10^4^ HHP-killed lung cancer cells [HHP], pulsed with HHP-killed cells and poly(I:C) [HHP+poly(I:C)] or stimulated with poly(I:C) only [poly(I:C)] for 24h. (A) The expression of CD80, CD86, CD83, HLA-DR and (E) CCR7 on CD11c^+^DAPI negative cells was assessed by flow cytometry. The values represent means ± SEM of 16 donors. Histograms are representative of 16 donors. (B) For phagocytic assays thawed HHP-killed H520 and H522 cells were stained with VybrantVR DiD and DC were stained with VybrantVR DiO at 37°C for 20 min before vaccine generation. The phagocytic capacity of DC was assessed as a percentage of DiO^+^DiD^+^ cells by flow cytometry after 24h. Graphs represent means ± SEM of n = 3 in duplicates. (C) Cytokine production was evaluated in cell culture supernatants after 24h by Luminex assay. Graphs represent means ± SEM of n = 5. (D) 3 × 10^5^ DC treated as described above were transferred to serum-containing media in upper chamber of transwell plate and were allowed to migrate to the lower chamber filled with media or media containing a mixture of CCL19 and CCL21 (both 50 ng/ml) at 37°C for 5h. The number of transmigrated CD11c^+^ DC was determined by flow cytometry. The graph shows mean ± SEM of 16 donors of n = 4. The results were considered statistically significant if * p < 0.05, ** p < 0.01 or *** p < 0.001.

To generate DC-based HHP lung cancer vaccine, DC were pulsed with a mixture of thawed HHP-killed H520 and H522 lung cancer cells for 4h and subsequently stimulated with poly(I:C). The phenotype of DC-based HHP lung cancer vaccine, its phagocytic capacity and chemotactic migration was assessed by flow cytometry after additional 20 h and was compared to immature DC, DC pulsed with HHP-killed cells alone or DC stimulated with poly(I:C) alone. Similarly, the production of pro-inflammatory cytokines was measured from cell culture supernatants by Luminex assay. As shown in Fig 1A, DC-based HHP lung cancer vaccine [HHP+poly(I:C)] displays significantly higher expression of CD80, CD83, HLA-DR and CD86 than control immature DC [iDC], DC pulsed with HHP-killed cells alone [HHP] or DC stimulated only with poly(I:C) [poly(I:C)]. Fig 1B shows that 80% of DC phagocytosed thawed HHP-killed lung cancer cells at 37°C after 24 h when compared to control DC incubated with HHP-killed cancer cells at 4°C. The addition of poly(I:C), however, decreased the phagocytic capacity of DC by 10% (Fig 1B). The slightly decreased phagocytosis of HHP-killed lung cancer cells by DC after addition of poly(I:C) did not affect the capacity of DC to produce pro-inflammatory cytokines such as IL-12p70, IFN-α and TNF-α (Fig 1C) or stimulate chemotactic migration (Fig 1D) and increase in CCR7 expression (Fig 1E). The production of IL-10 by DC-based HHP lung cancer vaccine was low and even significantly decreased when compared to IL-10 levels produced by DC treated with poly(I:C) only (Fig 1C). The immunogenicity of HHP-killed cells towards the DCs was confirmed [24] as DC pulsed with HHP-killed cells alone significantly enhanced DC maturation, CCR7 expression and the production of pro-inflammatory cytokines (Fig 1A, 1C and 1D).

In summary, these results showed that DC-based HHP lung cancer vaccine generated in serum-free media might be fully competent to stimulate T cell responses as shown by the efficient phagocytosis of HHP-killed lung cancer cells, an increased maturation and pro-inflammatory cytokine production and chemotactic migration.

### DC-based HHP lung cancer vaccine further increases its maturation and cytokine production in serum containing medium after additional LPS and CD40L stimulation

**Fig 2:**
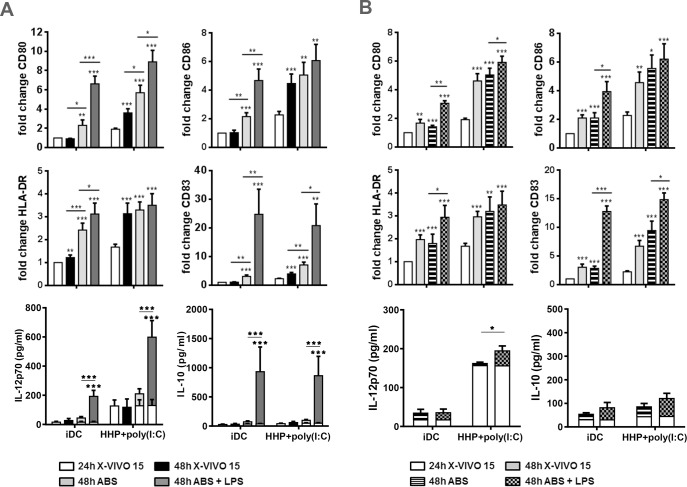
DC-based HHP lung cancer vaccine further increases its maturation and cytokine production in serum containing medium after additional LPS and CD40L stimulation. 2 × 10^5^ monocyte-derived DC generated in serum-free media X-VIVO 15 were left untreated [iDC] pulsed with thawed 4 × 10^4^ HHP-killed lung cancer cells and poly(I:C) [HHP+poly(I:C)] or stimulated with poly(I:C) only [poly(I:C)] for 24h [24h]. Serum-free medium was subsequently replaced with medium containing 10% human AB serum alone or supplemented with LPS (1 μg/ml) (A) or cells were loaded onto a layer of CD40L-expressiong A549 (B) for additional 24h [48h]. Non-transfected A549 cells were used as a negative control of CD40L stimulation (B). The difference in CD80, CD86, CD83 and HLA-DR expression between 24h and 48h samples was determined by flow cytometry. Similarly, the difference in IL-12p70 and IL-10 production was assessed by ELISA. Graphs show mean ± SEM of 8–18 donors of n = 5. The results were considered statistically significant if * p < 0.05, ** p < 0.01 or *** p < 0.001.

To assess further the plasticity and activity of DC-based HHP lung cancer vaccine in serum containing conditions and after additional stimulation, iDC and DC pulsed with HHP-killed cells and poly(I:C) for 24 h [24h] were transferred into RPMI 1640 containing 10% human AB serum for next 24 h [48h]. Cells were left untreated or treated with LPS or plated onto a layer of CD40L-transfected A459 cells. The expression of CD80, CD83, HLA-DR and CD86 was determined by flow cytometry. The production of IL-10 and IL-12p70 was assessed by ELISA. As shown in Fig 2A, the transfer of DC-based HHP lung cancer vaccine into serum containing conditions led to an increased expression of CD80 and CD83 within next 24 h compared to DC-based HHP lung cancer vaccine left in serum-free medium. LPS further enhanced the expression of CD80 and CD86 over the serum level. iDC incubated in serum free medium without HHP-killed tumor cell pulsation and poly(I:C) stimulation showed higher functional plasticity as all the maturation markers CD80, CD83, HLA-DR and CD86 increased after the transfer into serum-containing medium and were further significantly up-regulated over this level by LPS. However, the transfer of iDC or DC-based HHP lung cancer vaccine into serum containing medium did not induce IL-12p70 or IL-10 production. LPS, on the other hand, stimulated a significant production of both cytokines in the samples (Fig 2A).

Similar effect to LPS on DC maturation was observed after stimulation with CD40L-expressing A549 cells (Fig 2B). DC-based HHP lung cancer vaccine exhibited higher expression of CD80 and CD83 when stimulated with CD40L-expressing A549 in comparison to DC-based HHP lung cancer vaccine incubated with non-transfected A549. CD40L stimulation induced the expression of all markers CD80, CD83, HLA-DR and CD86 in iDC in contrast to stimulation with non-transfected A549 exhibiting again higher maturational plasticity than DC pulsed with HHP-killed tumor cells and poly(I:C).However, only DC pulsed with HHP-killed tumor cells and poly(I:C) were able to increase IL-12p70 production after CD40L stimulation (Fig 2B). In contrast to LPS, CD40L did not induce any IL-10 production in the samples. In conclusion, DC-based HHP lung cancer vaccine exhibits a capacity to be further stimulated and enhance its stimulatory potential.

### DC-based HHP lung cancer vaccine stimulates CD8^+^ and CD4^+^ T cells and decreases the number of CD4^+^CD25^+^Foxp3^+^T regulatory cells

**Fig 3:**
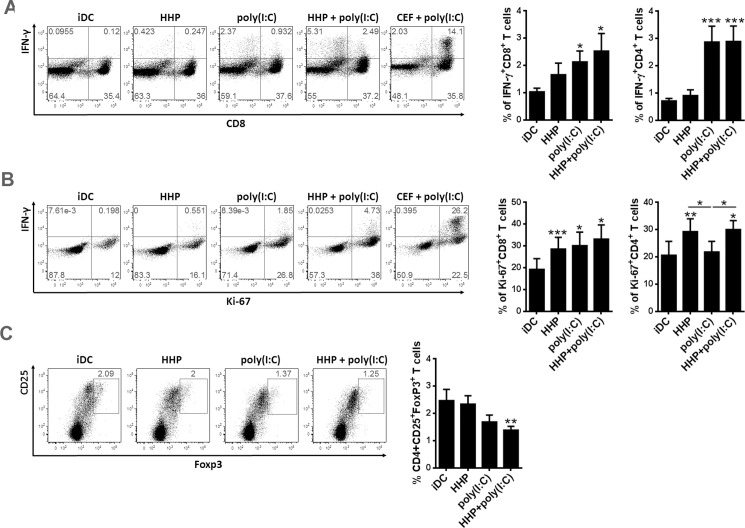
DC-based HHP lung cancer vaccine stimulates CD8^+^ and CD4^+^ T cells and decreases the number of CD4^+^CD25^+^Foxp3^+^T regulatory cells. 4 × 10^4^ iDC, DC pulsed with thawed HHP-killed lung cancer cells and poly(I:C) [HHP+poly(I:C)], or stimulated with poly(I:C) alone [poly(I:C)] for 24h were subsequently added to 2 ×10^5^ autologous T cells from healthy donors and subsequently co-cultured for 7 days. (A) IFN-γ-producing CD8^+^ and CD4^+^ T cells were assessed by flow cytometry after one round of restimulation with freshly prepared DC. CD8^+^ T cells shown in dotplots are gated from CD3^+^ cells, and predominantly CD4^+^ T cells are displayed as CD8 negative cells. The percentage in graphs is displayed as a percentage of IFN-γ-producing cells from CD8^+^ or CD4^+^ T cells, respectively, therefore it does not correspond to percentages in dotplots. (B) T cell proliferation (Ki-67) and (C) the number of CD4^+^CD25^+^FoxP3^+^T regulatory cells was determined without restimulation by flow cytometry. Dotplots are representative of 8 donors. Graphs represent means ± SEM of 8 donors from n = 3. The results were considered statistically significant if * p < 0.05, ** p < 0.01 or *** p < 0.001.

We further examine if DC-based HHP lung cancer vaccine induces proliferation and IFN-γ-production in CD8^+^ and CD4^+^ T cells. After 24h iDC, DC pulsed with HHP-killed tumor cells alone, DC pulsed with HHP-killed tumor cells and poly(I:C) or DC stimulated with poly(I:C) only were co-cultured with autologous T lymphocytes at ratio 1:5 for 7 days. T cell proliferation (Ki-67) and IFN-γ production was measured by flow cytometry after restimulation with corresponding freshly prepared DC. As shown in graphs in Fig 3A, DC-based HHP lung cancer vaccine stimulated significantly higher number of IFN-γ-producing CD8^+^ and CD4^+^ T cells in comparison to iDC or DC pulsed with HHP-killed tumor cells alone. However, IFN-γ-producing CD8^+^ and CD4^+^ T cells were also significantly stimulated by DC treated only with poly(I:C) similarly to DC-based HHP lung cancer vaccine. High percentage of IFN-γ producing CD8^+^ T cells was detected after stimulation with poly(I:C)-stimulated DC loaded with a mixture of antigenic peptides to CMV, EBV and influenza [CEF+poly(I:C)] which was used as a positive control (Fig 3A). Whereas no differences were observed in the induction of IFN-γ-production in T cells between DC-based lung cancer vaccine and DC-treated only with poly(I:C), we observed a significant differences in T cell proliferation. DC pulsed with HHP-killed cells alone induced high CD8^+^ and CD4^+^ T cell proliferation (Fig 3B).Which was not further enhanced by addition of poly(I:C). DC treated with poly(I:C) alone stimulated similar proliferation of CD8^+^ T cells, however failed to induce the proliferation of CD4^+^ T cells. These results indicate that DC-based HHP lung cancer vaccine stimulates T cells, and might be specifically efficient in inducing CD4^+^ T cell proliferation. Next we addressed the number of CD4^+^CD25^+^Foxp3^+^ T regulatory cells induced by DC-based HHP lung cancer vaccine after 7 days. Fig 3C shows that there was a significantly lower number of CD4^+^CD25^+^Foxp3^+^ T regulatory cells induced by DC-based HHP lung cancer vaccine when compared to iDC and DC pulsed with HHP-killed tumor cells alone. Poly(I:C) stimulation alone decreased the number of CD4^+^CD25^+^Foxp3^+^ T regulatory cells, however the number of CD4^+^CD25^+^Foxp3^+^ T regulatory cell was still slightly higher than the number of T regulatory cells induced by the DC-based lung cancer vaccine (Fig 3C).

### DC-based HHP lung cancer vaccine generated from NSCLC patients’ monocytes induces tumor-antigen specific CD8^+^ and CD4^+^ T cells

**Fig 4:**
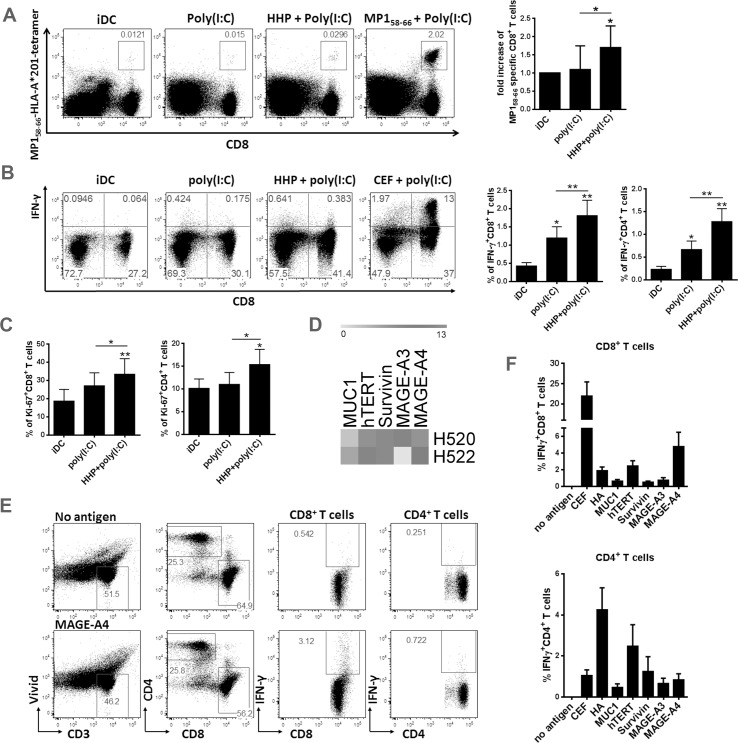
DC-based HHP lung cancer vaccine generated from NSCLC patients’ monocytes induces tumor-antigen specific CD8^+^ and CD4^+^ T cells. 4 × 10^4^ iDC, DC pulsed with thawed HHP-killed lung cancer cells or MP1-expressing A549 and poly(I:C) [HHP+poly(I:C)] or stimulated with poly(I:C) alone [poly(I:C)] for 24h were subsequently added to 2 × 10^5^ autologous T cells from NSCLC patients and subsequently co-cultured for 7 days. (A) The MP1_58-66_-specific CD8^+^ T cells were detected without restimulation with MP1_58-66_-HLA-A*201 Tetramer staining by flow cytometry. Dotplots are representative of 4 donors. Graphs represent means ± SEM of 4 donors from n = 2. (B) IFN-γ-producing CD8^+^ and CD4^+^ T cells were assessed by flow cytometry after one round of restimulation with freshly prepared NSCLC patient’ DC. The percentage in graphs is displayed as a percentage of IFN-γ-producing cells from CD8^+^ or CD4^+^ T cells, respectively, therefore it does not correspond to percentages in dotplots. (C) T cell proliferation (Ki-67) was determined without restimulation by flow cytometry. Dotplots are representative of 6 patients. Graphs show means of ± SEM of 6 donors in duplicates. The results were considered statistically significant if * p < 0.05, ** p < 0.01 or *** p < 0.001. (D) qPCR analyses of Mucin-1, hTERT, Survivin, MAGE-A3 and MAGE-A4 expression in H520 and H522 cell lines (n = 3, in duplicates) (E) Gating strategy to detect tumor antigen specific IFN-γ producing T cells in NSCLC patients. One representative patient sample for MAGE-A4-specific T cells is shown. (F) Quantitative evaluation of tumor antigen specific IFN-γ producing T cells in 36 NLCLC patients. The percentage of negative control (no antigen) was deducted from the samples stimulated with antigenic pepmixes.

Our results indicate that DC-based HHP lung cancer vaccine stimulates T cells. However, given no differences between DC-based HHP lung cancer vaccine and DC stimulated with poly(I:C) alone, it is not clear if induced T cells recognize antigens which come from the HHP-killed lung cancer cells. To test this, we generated a model antigenic system encompassing A549 cell line expressing a short version of matrix protein 1 (MP1) of influenza virus. Autologous T cells were stimulated with HLA-A*201^+^ iDC, DC stimulated with poly(I:C) alone or DC loaded with thawed HHP-killed A549 expressing MP1 and poly(I:C) for 8 days (Fig 4A). The number of MP1_58-66_-specific CD8^+^ T cells was detected with MP1_58-66_-HLA-A*201 Tetramer staining by flow cytometry. As shown in Fig 4A DC-based HHP lung cancer vaccine stimulated the expansion of MP1_58-66_-specific CD8^+^ T cells over iDC or DC treated with poly(I:C) alone which proves that protein antigens from HHP-killed lung cancer cells are processed and presented by DC to T cells. Next we validated our results on DC-based HHP lung cancer vaccine generated from several NSCLC patients. DCs were generated in serum-free media from CD14^+^ isolated monocytes. Autologous T lymphocytes were stimulated and restimulated with fresh patient’s DC as described above. As shown in Fig 4B, DC-based HHP lung cancer vaccine generated from PBMCs of NSCLC patients stimulated significantly higher amount of IFN-γ-producing CD8^+^ and CD4^+^ T cells than DC stimulated with poly(I:C) alone or iDC. Similarly, higher proliferation of CD8^+^ and CD4^+^ T cells was detected after DC-based HHP lung cancer vaccine stimulation (Fig 4C). These results showed that DC-based HHP lung cancer vaccine stimulates tumor-antigen specific IFN-γ-producing T cells present in NSCLC patient’s blood *in vitro*. To show that tumor-specific CD8^+^ and CD4^+^ T cell responses to tumor antigens expressed specifically in H522 and H520 lung cancer cell lines are present in NSCLC patients we analyzed the expression of MAGE-A3, MAGE-A4, hTERT, Survivin and Mucin-1, well-characterized antigens found in lung cancer tumors [27–29] by qPCR. Various degree of expression of all antigens, except of MAGE-A3 in H522, was detected in both cell lines (Fig 4D). The presence of CD8^+^ and CD4^+^ T cell responses specific to these antigens was analyzed by stimulation of PBMCs from 36 NSCLC patients with corresponding mixture of antigenic peptides. One representative donor responsive to MAGE-A4 pepmixes stimulation is shown in Fig 4E. In 36 NSCLC patients we detected IFN-γ-producing CD8^+^ and CD4^+^ T cells responsive to the all tested antigens (Fig 4F) which support our findings that our DC-based HHP lung cancer vaccine is fully competent to induce anti-tumor T cell responses in NSCLC patients.

## Discussion

DC-based active cellular immunotherapy represents a large field of immunotherapeutic approaches to cancer treatment; however its use is quite limited in immunotherapy of lung carcinoma. In our study we showed that DC-based lung cancer vaccine generated in GMP-complaint serum free media with HHP-killed lung cancer cell lines and poly(I:C) exhibits functional plasticity and activity to stimulate tumor antigen-specific IFN-γ-producing CD8^+^ and CD4^+^ T cells from NSCLC patients.

HHP-killed lung cancer cell lines used for the vaccine generation in this study consisted of allogeneic commercially available adenocarcinoma (H522) and squamous cell carcinoma (H520) cell lines. These cell lines cover 80% of NSCLC histological subtypes and are used as a source of broad range of tumor-associated antigens (TAA) which were shown to be differentially expressed in NSCLC patients [27, 30, 28]. Although DC-based vaccines pulsed with TAA-derived peptides have been shown to induce immune response in NSCLC patients [9, 8, 7, 10], broader TAA availability provided by HHP-killed cell lines might induce immune responses in higher number of patients. The other advantage of allogeneic cell lines is the possibility to cultivate them in high numbers in contrast to autologous tumor material which might be difficult to obtain in sufficient amount due to a frequent inoperability of late stages of the disease in NSCLC patients. Interestingly, only one research group have so far used irradiated and UBV-killed allogeneic adenocarcinoma cell line 1650 for DC-based vaccine generation in phase I clinical trials [16, 15, 18, 17]. The immune responses were observed in 6 out 11 of immunized patients as detected by IFN-γ ELISPOT assay [18]. This shows that killed allogeneic cell lines are safe and suitable for DC-based active lung cancer immunotherapy.

In this study, we showed that HHP treatment generates immunogenic cancer cells, similarly to our previously published data [24]. Phagocytosis of HHP-killed tumor cells by DC stimulated the expression of maturation-associated molecules on DCs and induced production pro-inflammatory cytokines. However, our observed effects on DCs maturation and cytokine production were lower than previously published [24]. This can be explained by DC cultivation in serum free media in our study in contrast to serum-containing media, which represents optimal cultivation conditions for the DC, in the previous study [24]. Poly(I:C)-matured DC exhibit the best T cell stimulatory capacity *in vitro* [31]. In our study, DC pulsed with HHP-killed lung cancer cells and poly(I:C) expressed even higher levels of maturation-associated molecules than DC stimulated with poly(I:C) only which suggests a synergistic stimulatory effect of phagocytosed immunogenic HHP-killed cells and poly(I:C). The slight decrease in phagocytic capacity of DC after addition of poly(I:C) could be explained by the induction of DC maturation which is accompanied by the reduction in antigen uptake as DC concomitantly increase their antigen processing and presentation capacity [4, 32]. The increased chemotactic migration and pro-inflammatory cytokine production was, on the other hand, comparable between poly(I:C)-stimulated DC and DC-based HHP lung cancer vaccine. This suggests that immunogenicity of HHP-killed cells did not contribute to cytokine production or chemotactic migration induced by poly(I:C). Low IL-10 production and high IL-12p70, TNF-α and IFN-α confirm Th1 polarizing properties of DC induced by poly(I:C).

We also showed that DC-based HHP lung cancer vaccine exhibited functional plasticity after transfer into serum containing conditions which would simulate the transfer of the vaccine into NSCLC patients. DC-based HHP lung cancer vaccine was not functionally exhausted by the first maturation stimuli with poly(I:C) as DC enhanced the expression of maturation associated molecules CD80 and CD83 and IL-12p70 production in response to LPS and CD40L [4, 33, 34]. LPS represent a strong maturation signal which is not likely to occur in NSCLC patients unless there is a bacterial infection. On the other hand, CD40L stimulation represent a physiological signaling which DC-based HHP lung cancer vaccine is likely to encounter on the surface of other immune cells such as B and T cells after administration into NSCLC patients. CD40-CD40L ligation provides necessary costimulatory signals for B and T cells, it is important for induction of cytotoxic CD8^+^ T lymphocytes and also ensures long-term survival of DC [35, 36, 33]. In keeping with our data, it has been previously reported that human DC matured with poly(I:C) retained their capacity to produce IL-12p70 after additional CD40L stimulation [37]. Moreover CD40L ligation enhances DC cross-presentation [38]. Our data therefore suggest that DC-based HHP lung cancer vaccine might gain further stimulatory activity *in vivo* after administration into NSCLC patients.

Most importantly, we observed that DC-based HHP lung cancer vaccine stimulates proliferation and IFN-γ production in CD8^+^ and CD4^+^ T cells and lowers the numbers of CD4^+^CD25^+^Foxp3^+^ T regulatory cells *in vitro*. The T cell stimulatory capacity of DC-based HHP lung cancer vaccine generated from healthy donors was, however, comparable to that of DC stimulated with poly(I:C) alone which indicates that T cells which were not specific to TAA derived from HHP-killed lung cancer cells were induced. As the detection of TAA-specific T cells, specifically by single epitope tetramers, is technically challenging due to their low frequency in blood (10^−7^) [39, 40], we generated an artificial antigenic system based on an immunodominant CD8^+^ T cell epitope from influenza MP1 protein. The frequency of influenza-specific CD8^+^ T cells can range between 10^−2^ to10^-4^ [41]. By using MP1-expressing A549 cells we were able to prove in HLA-A*201^+^ healthy donors that protein antigen derived from HHP-killed lung cancer cells is indeed processed and presented by DC to autologous CD8^+^ T cells. Moreover, using DC-based HHP lung cancer vaccine generated from NSCLC patients we observed an increase in proliferation and IFN-γ production in CD8^+^ and CD4^+^ T cells over the T cells stimulated with patient’s DC-treated with poly(I:C) only. This indicates that TAA-specific T cells were stimulated. We have confirmed here the presence of tumor antigen-specific T cells in the blood of NSCLC patients against selected antigens commonly found in lung cancer [27, 29, 28]. We showed that these antigens are also expressed in lung cancer cell lines H520 and H522 used in this study to generate DC-based HHP lung cancer vaccine. Several clinical trials with peptide or protein vaccine targeted against these antigens have shown the induction of antigen specific T cells and also a clinical benefit in NSCLC patients [42–44]. This is supportive of DC-based HHP lung cancer vaccine effectivity in NSCLC patients. The role of CD8^+^ T cells in anticancer immunity is well established, however, it is currently not clear if CD4^+^ T cells induced by DC-based HHP lung cancer vaccine can contribute to or mediate significant anti-tumor effect [45]. As we observed a high proliferation of CD4^+^ T cells induced by DC-based lung cancer vaccine, it is tempting to speculate that CD4^+^ T cells might play an important role in shaping anti-cancer responses after DC-based HHP lung cancer immunotherapy in NSCLC patients. DC-based HHP cancer vaccine alone or in combination with chemotherapy was shown to significantly inhibited growth of tumors in mouse models [25], however the differential contribution of CD8^+^ or CD4^+^T cells was not analyzed.

In conclusion we showed that DC-based HHP lung cancer vaccine generated in GMP compliant serum free medium is immunologically active and displays functional plasticity towards immunostimulatory Th1 polarizing phenotype *in vitro*. These results represent important preclinical data for the ongoing Phase I/II clinical trial combining DC-based active immunotherapy (DCVAC/LuCa) with chemotherapy and immune enhancers (NCT02470468).

## Supporting information

S1 Fig The induction of cell death and the exposure of immunogenic molecules (HSP70, HSP90 and calreticulin) by HHP in H520 and H522. (A) 1×10^6^ cells/ml of H520 a H522 were treated with HHP 250 MPa for 10 min, incubated at 37°C for 2h and subsequently frozen at -80°C for 24h. Cell viability of non-treated cells, HHP-killed cells before freezing [2h] and after thawing [2h+24h] was determined with Annexin-PE and DAPI staining using flow cytometry. Dotplots show one representative experiment. Graph shows means ± SEM of n = 5 in duplicates. (B) Exposure of HSP70, HSP90 and calreticulin was determined by flow cytometry. Graphs represent means ± SEM of n = 3 in duplicates.
(TIF)

## References

[1] Mellstedt H, Vansteenkiste J, Thatcher N. Vaccines for the treatment of non-small cell lung cancer: investigational approaches and clinical experience. Lung cancer. 2011;73(1):11–7. 10.1016/j.lungcan.2011.02.023 21474197

[2] Skachkova OV, Khranovska NM, Gorbach OI, Svergun NM, Sydor RI, Nikulina VV. Immunological markers of anti-tumor dendritic cells vaccine efficiency in patients with non-small cell lung cancer. Experimental oncology. 2013;35(2):109–13. 23828386

[3] Datta J, Terhune JH, Lowenfeld L, Cintolo JA, Xu S, Roses RE et al Optimizing dendritic cell-based approaches for cancer immunotherapy. The Yale journal of biology and medicine. 2014;87(4):491–518. 25506283 PMC4257036

[4] Banchereau J, Briere F, Caux C, Davoust J, Lebecque S, Liu YJ et al Immunobiology of dendritic cells. Annual review of immunology. 2000;18:767–811. 10.1146/annurev.immunol.18.1.767 10837075

[5] Mukherji B, Chakraborty NG, Yamasaki S, Okino T, Yamase H, Sporn JR et al Induction of antigen-specific cytolytic T cells in situ in human melanoma by immunization with synthetic peptide-pulsed autologous antigen presenting cells. Proceedings of the National Academy of Sciences of the United States of America. 1995;92(17):8078–82. 7644541 10.1073/pnas.92.17.8078 PMC41290

[6] Sheikh NA, Petrylak D, Kantoff PW, Dela Rosa C, Stewart FP, Kuan LY et al Sipuleucel-T immune parameters correlate with survival: an analysis of the randomized phase 3 clinical trials in men with castration-resistant prostate cancer. Cancer immunology, immunotherapy: CII. 2013;62(1):137–47. 10.1007/s00262-012-1317-2 22865266 PMC3541926

[7] Ueda Y, Itoh T, Nukaya I, Kawashima I, Okugawa K, Yano Y et al Dendritic cell-based immunotherapy of cancer with carcinoembryonic antigen-derived, HLA-A24-restricted CTL epitope: Clinical outcomes of 18 patients with metastatic gastrointestinal or lung adenocarcinomas. International journal of oncology. 2004;24(4):909–17. 15010829

[8] Perroud MW Jr., Honma HN, Barbeiro AS, Gilli SC, Almeida MT, Vassallo J et al Mature autologous dendritic cell vaccines in advanced non-small cell lung cancer: a phase I pilot study. Journal of experimental & clinical cancer research: CR. 2011;30:65. 21682877 10.1186/1756-9966-30-65 PMC3135553

[9] Babatz J, Rollig C, Lobel B, Folprecht G, Haack M, Gunther H et al Induction of cellular immune responses against carcinoembryonic antigen in patients with metastatic tumors after vaccination with altered peptide ligand-loaded dendritic cells. Cancer immunology, immunotherapy: CII. 2006;55(3):268–76. 10.1007/s00262-005-0021-x 16034561 PMC11031026

[10] Takahashi H, Okamoto M, Shimodaira S, Tsujitani S, Nagaya M, Ishidao T et al Impact of dendritic cell vaccines pulsed with Wilms' tumour-1 peptide antigen on the survival of patients with advanced non-small cell lung cancers. European journal of cancer. 2013;49(4):852–9. 10.1016/j.ejca.2012.11.005 23245331

[11] Morse MA, Clay TM, Hobeika AC, Osada T, Khan S, Chui S et al Phase I study of immunization with dendritic cells modified with fowlpox encoding carcinoembryonic antigen and costimulatory molecules. Clinical cancer research: an official journal of the American Association for Cancer Research. 2005;11(8):3017–24. 15837756 10.1158/1078-0432.CCR-04-2172

[12] Kontani K, Taguchi O, Ozaki Y, Hanaoka J, Sawai S, Inoue S et al Dendritic cell vaccine immunotherapy of cancer targeting MUC1 mucin. International journal of molecular medicine. 2003;12(4):493–502. 12964025

[13] Chang GC, Lan HC, Juang SH, Wu YC, Lee HC, Hung YM et al A pilot clinical trial of vaccination with dendritic cells pulsed with autologous tumor cells derived from malignant pleural effusion in patients with late-stage lung carcinoma. Cancer. 2005;103(4):763–71. 10.1002/cncr.20843 15637694

[14] Engell-Noerregaard L, Hendel HW, Johannesen HH, Alslev L, Svane IM. FDG PET scans as evaluation of clinical response to dendritic cell vaccination in patients with malignant melanoma. Cancer immunology, immunotherapy: CII. 2013;62(1):17–25. 10.1007/s00262-012-1306-5 22722450 PMC11029132

[15] Hirschowitz EA, Foody T, Kryscio R, Dickson L, Sturgill J, Yannelli J. Autologous dendritic cell vaccines for non-small-cell lung cancer. Journal of clinical oncology: official journal of the American Society of Clinical Oncology. 2004;22(14):2808–15. 15254048 10.1200/JCO.2004.01.074

[16] Hirschowitz EA, Foody T, Hidalgo GE, Yannelli JR. Immunization of NSCLC patients with antigen-pulsed immature autologous dendritic cells. Lung cancer. 2007;57(3):365–72. 10.1016/j.lungcan.2007.04.002 17509725 PMC2063443

[17] Yannelli JR, Sturgill J, Foody T, Hirschowitz E. The large scale generation of dendritic cells for the immunization of patients with non-small cell lung cancer (NSCLC). Lung cancer. 2005;47(3):337–50. 10.1016/j.lungcan.2004.08.008 15713517

[18] Hirschowitz EA, Mullins A, Prajapati D, Baeker T, Kloecker G, Foody T et al Pilot study of 1650-G: a simplified cellular vaccine for lung cancer. Journal of thoracic oncology: official publication of the International Association for the Study of Lung Cancer. 2011;6(1):169–73. 10.1097/JTO.0b013e3181fb5c22 21150468

[19] Um SJ, Choi YJ, Shin HJ, Son CH, Park YS, Roh MS et al Phase I study of autologous dendritic cell tumor vaccine in patients with non-small cell lung cancer. Lung cancer. 2010;70(2):188–94. 10.1016/j.lungcan.2010.02.006 20223553

[20] Kroemer G, Galluzzi L, Kepp O, Zitvogel L. Immunogenic cell death in cancer therapy. Annual review of immunology. 2013;31:51–72. 10.1146/annurev-immunol-032712-100008 23157435

[21] Adkins I, Fucikova J, Garg AD, Agostinis P, Spisek R. Physical modalities inducing immunogenic tumor cell death for cancer immunotherapy. Oncoimmunology. 2014;3(12):e968434 10.4161/21624011.2014.968434 25964865 PMC4352954

[22] Tesniere A, Panaretakis T, Kepp O, Apetoh L, Ghiringhelli F, Zitvogel L et al Molecular characteristics of immunogenic cancer cell death. Cell death and differentiation. 2008;15(1):3–12. 10.1038/sj.cdd.4402269 18007663

[23] Krysko DV, Garg AD, Kaczmarek A, Krysko O, Agostinis P, Vandenabeele P. Immunogenic cell death and DAMPs in cancer therapy. Nature reviews Cancer. 2012;12(12):860–75. 10.1038/nrc3380 23151605

[24] Fucikova J, Moserova I, Truxova I, Hermanova I, Vancurova I, Partlova S et al High hydrostatic pressure induces immunogenic cell death in human tumor cells. International journal of cancer Journal international du cancer. 2014;135(5):1165–77. 10.1002/ijc.28766 24500981

[25] Mikyskova R, Stepanek I, Indrova M, Bieblova J, Simova J, Truxova I et al Dendritic cells pulsed with tumor cells killed by high hydrostatic pressure induce strong immune responses and display therapeutic effects both in murine TC-1 and TRAMP-C2 tumors when combined with docetaxel chemotherapy. International journal of oncology. 2016;48(3):953–64. 10.3892/ijo.2015.3314 26718011 PMC4750542

[26] Truxova I, Pokorna K, Kloudova K, Partlova S, Spisek R, Fucikova J. Day 3 Poly (I:C)-activated dendritic cells generated in CellGro for use in cancer immunotherapy trials are fully comparable to standard Day 5 DCs. Immunology letters. 2014;160(1):39–49. 10.1016/j.imlet.2014.03.010 24726860

[27] Gure AO, Chua R, Williamson B, Gonen M, Ferrera CA, Gnjatic S et al Cancer-testis genes are coordinately expressed and are markers of poor outcome in non-small cell lung cancer. Clinical cancer research: an official journal of the American Association for Cancer Research. 2005;11(22):8055–62. 16299236 10.1158/1078-0432.CCR-05-1203

[28] Karanikas V, Tsochas S, Boukas K, Kerenidi T, Nakou M, Dahabreh J et al Co-expression patterns of tumor-associated antigen genes by non-small cell lung carcinomas: implications for immunotherapy. Cancer biology & therapy. 2008;7(3):345–52. 18094614 10.4161/cbt.7.3.5424

[29] Tajima K, Obata Y, Tamaki H, Yoshida M, Chen YT, Scanlan MJ et al Expression of cancer/testis (CT) antigens in lung cancer. Lung cancer. 2003;42(1):23–33. 14512184 10.1016/s0169-5002(03)00244-7

[30] Grunwald C, Koslowski M, Arsiray T, Dhaene K, Praet M, Victor A et al Expression of multiple epigenetically regulated cancer/germline genes in nonsmall cell lung cancer. International journal of cancer Journal international du cancer. 2006;118(10):2522–8. 10.1002/ijc.21669 16353146

[31] Fucikova J, Rozkova D, Ulcova H, Budinsky V, Sochorova K, Pokorna K et al Poly I: C-activated dendritic cells that were generated in CellGro for use in cancer immunotherapy trials. Journal of translational medicine. 2011;9:223 10.1186/1479-5876-9-223 22208910 PMC3259090

[32] West MA, Wallin RP, Matthews SP, Svensson HG, Zaru R, Ljunggren HG et al Enhanced dendritic cell antigen capture via toll-like receptor-induced actin remodeling. Science. 2004;305(5687):1153–7. 10.1126/science.1099153 15326355

[33] Macagno A, Napolitani G, Lanzavecchia A, Sallusto F. Duration, combination and timing: the signal integration model of dendritic cell activation. Trends in immunology. 2007;28(5):227–33. 10.1016/j.it.2007.03.008 17403614

[34] Abdi K, Singh NJ, Matzinger P. Lipopolysaccharide-activated dendritic cells: "exhausted" or alert and waiting? Journal of immunology. 2012;188(12):5981–9. 10.4049/jimmunol.1102868 PMC3370068 22561154

[35] Cella M, Scheidegger D, Palmer-Lehmann K, Lane P, Lanzavecchia A, Alber G. Ligation of CD40 on dendritic cells triggers production of high levels of interleukin-12 and enhances T cell stimulatory capacity: T-T help via APC activation. The Journal of experimental medicine. 1996;184(2):747–52. 8760829 10.1084/jem.184.2.747 PMC2192696

[36] Ma DY, Clark EA. The role of CD40 and CD154/CD40L in dendritic cells. Seminars in immunology. 2009;21(5):265–72. 10.1016/j.smim.2009.05.010 19524453 PMC2749083

[37] Rouas R, Lewalle P, El Ouriaghli F, Nowak B, Duvillier H, Martiat P. Poly(I:C) used for human dendritic cell maturation preserves their ability to secondarily secrete bioactive IL-12. International immunology. 2004;16(5):767–73. 10.1093/intimm/dxh077 15096480

[38] Delamarre L, Holcombe H, Mellman I. Presentation of exogenous antigens on major histocompatibility complex (MHC) class I and MHC class II molecules is differentially regulated during dendritic cell maturation. The Journal of experimental medicine. 2003;198(1):111–22. 10.1084/jem.20021542 12835477 PMC2196081

[39] Alanio C, Lemaitre F, Law HK, Hasan M, Albert ML. Enumeration of human antigen-specific naive CD8+ T cells reveals conserved precursor frequencies. Blood. 2010;115(18):3718–25. 10.1182/blood-2009-10-251124 20200354

[40] Coulie PG, Connerotte T. Human tumor-specific T lymphocytes: does function matter more than number? Current opinion in immunology. 2005;17(3):320–5. 10.1016/j.coi.2005.03.002 15886124

[41] He XS, Mahmood K, Maecker HT, Holmes TH, Kemble GW, Arvin AM et al Analysis of the frequencies and of the memory T cell phenotypes of human CD8+ T cells specific for influenza A viruses. The Journal of infectious diseases. 2003;187(7):1075–84. 10.1086/368218 12660922

[42] Takahashi N, Ohkuri T, Homma S, Ohtake J, Wakita D, Togashi Y et al First clinical trial of cancer vaccine therapy with artificially synthesized helper/ killer-hybrid epitope long peptide of MAGE-A4 cancer antigen. Cancer science. 2012;103(1):150–3. 10.1111/j.1349-7006.2011.02106.x 22221328 PMC11164142

[43] Atanackovic D, Altorki NK, Stockert E, Williamson B, Jungbluth AA, Ritter E et al Vaccine-induced CD4+ T cell responses to MAGE-3 protein in lung cancer patients. Journal of immunology. 2004;172(5):3289–96. 10.4049/jimmunol.172.5.3289 14978137

[44] Brunsvig PF, Kyte JA, Kersten C, Sundstrom S, Moller M, Nyakas M et al Telomerase peptide vaccination in NSCLC: a phase II trial in stage III patients vaccinated after chemoradiotherapy and an 8-year update on a phase I/II trial. Clinical cancer research: an official journal of the American Association for Cancer Research. 2011;17(21):6847–57. 21918169 10.1158/1078-0432.CCR-11-1385

[45] Perez-Diez A, Joncker NT, Choi K, Chan WF, Anderson CC, Lantz O et al CD4 cells can be more efficient at tumor rejection than CD8 cells. Blood. 2007;109(12):5346–54. 10.1182/blood-2006-10-051318 17327412 PMC1890845

