# Supplementary figures and images for: Generation of dendritic cell-based vaccine using high hydrostatic pressure for non-small cell lung cancer immunotherapy
Source: PLoS One. 2017 Feb 10;12(2):e0171539. doi: 10.1371/journal.pone.0171539 (PMC5302789; doi:10.1371/journal.pone.0171539)

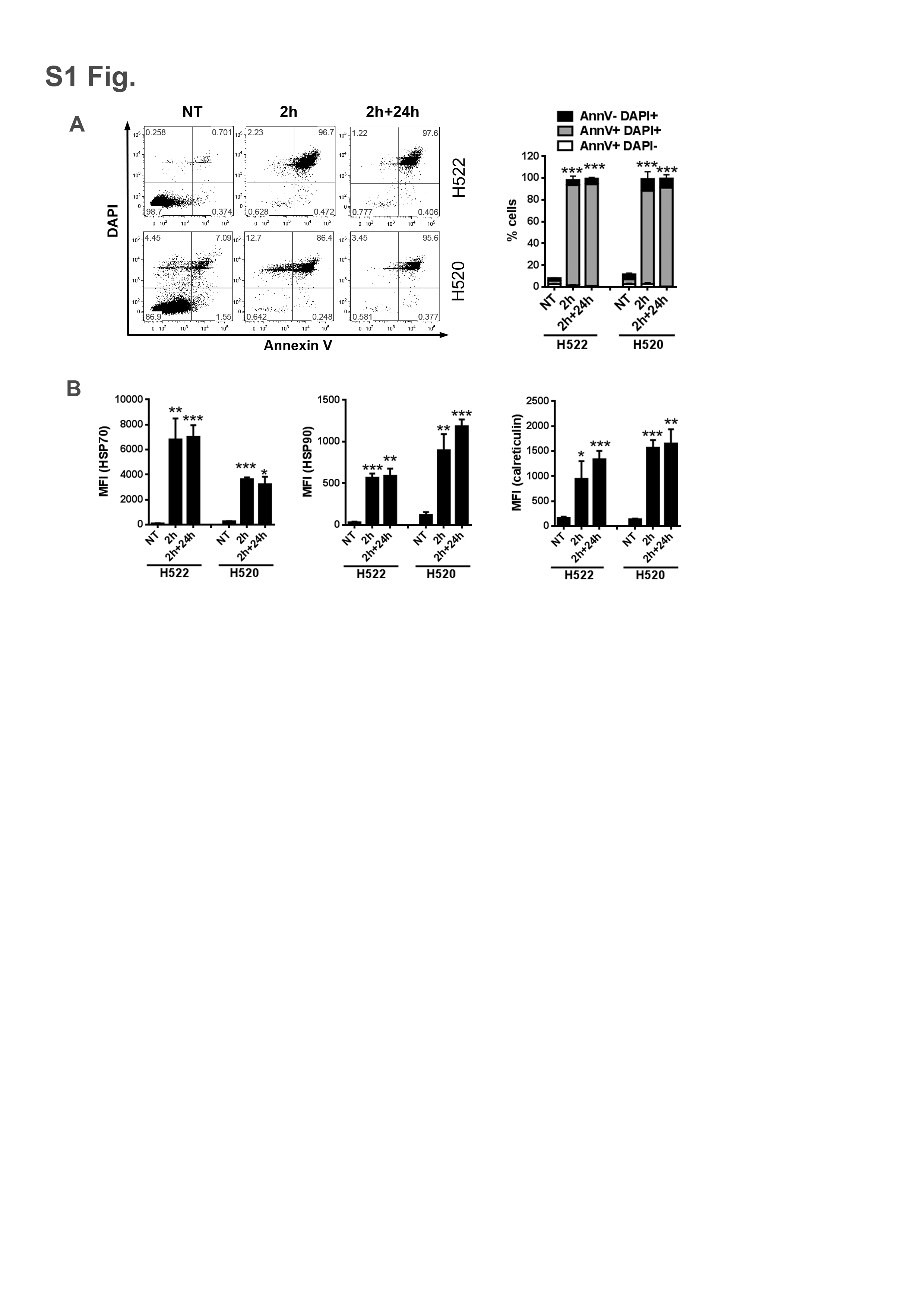

Supplement: S1 Fig — (A) 1×10^6^ cells/ml of H520 a H522 were treated with HHP 250 MPa for 10 min, incubated at 37°C for 2h and subsequently frozen at -80°C for 24h. Cell viability of non-treated cells, HHP-killed cells before freezing [2h] and after thawing [2h+24h] was determined with Annexin-PE and DAPI staining using flow cytometry. Dotplots show one representative experiment. Graph shows means ± SEM of n = 5 in duplicates. (B) Exposure of HSP70, HSP90 and calreticulin was determined by flow cytometry. Graphs represent means ± SEM of n = 3 in duplicates. (TIF) [file pone.0171539.s001.tif]
